# Leptin signalling regulates transcriptional differences in granulosa cells from genetically obese mice but not the activation of NLRP3 inflammasome

**DOI:** 10.1038/s41598-024-58181-w

**Published:** 2024-04-05

**Authors:** Marek Adamowski, Yashaswi Sharma, Tomasz Molcan, Karolina Wołodko, Gavin Kelsey, António M. Galvão

**Affiliations:** 1 Department of Reproductive Immunology and Pathology, Institute of Animal Reproduction and Food Research of Polish Academy of Sciences, Olsztyn, Poland; 2 Epigenetics Programme, The Babraham Institute, Cambridge, CB22 3AT UK; 3 Centre for Trophoblast Research, University of Cambridge, Cambridge, CB2 3EG UK; 4 Department of Comparative Biomedical Sciences, Royal Veterinary College, 4 Royal College Street, London, NW1 0TU UK

**Keywords:** Leptin, Obesity, Ovary, Macrophage, NLRP3 inflammasome, Reproductive biology, Mechanisms of disease, Ovary, Experimental models of disease

## Abstract

Obesity is associated with increased ovarian inflammation and the establishment of leptin resistance. We presently investigated the role of impaired leptin signalling on transcriptional regulation in granulosa cells (GCs) collected from genetically obese mice. Furthermore, we characterised the association between ovarian leptin signalling, the activation of the NOD-like receptor protein 3 (NLRP3) inflammasome and macrophage infiltration in obese mice. After phenotype characterisation, ovaries were collected from distinct group of animals for protein and mRNA expression analysis: (i) mice subjected to a diet-induced obesity (DIO) protocol, where one group was fed a high-fat diet (HFD) and another a standard chow diet (CD) for durations of 4 or 16 weeks; (ii) mice genetically deficient in the long isoform of the leptin receptor (ObRb; db/db); (iii) mice genetically deficient in leptin (ob/ob); and (iv) mice rendered pharmacologically hyperleptinemic (LEPT). Next, GCs from antral follicles isolated from *db/db* and *ob/ob* mice were subjected to transcriptome analysis. Transcriptional analysis revealed opposing profiles in genes associated with steroidogenesis and prostaglandin action between the genetic models, despite the similarities in body weight. Furthermore, we observed no changes in the mRNA and protein levels of NLRP3 inflammasome components in the ovaries of *db/db* mice or in markers of M1 and M2 macrophage infiltration. This contrasted with the downregulation of NLRP3 inflammasome components and M1 markers in *ob/ob* and 16-wk HFD-fed mice. We concluded that leptin signalling regulates NLRP3 inflammasome activation and the expression of M1 markers in the ovaries of obese mice in an ObRb-dependent and ObRb-independent manner. Furthermore, we found no changes in the expression of leptin signalling and NLRP3 inflammasome genes in GCs from *db/db* and *ob/ob* mice, which was associated with no effects on macrophage infiltration genes, despite the dysregulation of genes associated with steroidogenesis in homozygous obese *db/db*. Our results suggest that: (i) the crosstalk between leptin signalling, NLRP3 inflammasome and macrophage infiltration takes place in ovarian components other than the GC compartment; and (ii) transcriptional changes in GCs from homozygous obese *ob/ob* mice suggest structural rearrangement and organisation, whereas in *db/db* mice the impairment in steroidogenesis and secretory activity.

## Introduction

Obesity is recurrently associated with several comorbidities like diabetes, cardiovascular disease and dyslipidaemia^1^. Importantly, obesity also affects fertility^2,3^. Obese women present impaired ovulation, decreased oocyte quality and higher levels of embryonic arrest^4^. Overall, increased maternal body mass index (BMI) is a known risk factor for foetal death, stillbirth and perinatal and infant death^5,6^. At ovarian level, studies on granulosa cells (GCs) from obese women have shown increased mitochondrial damage, endoplasmic reticulum stress, and impaired steroidogenesis^7^. Ultimately, oocytes from obese mothers present higher levels of reactive oxygen species, oxidative stress, apoptosis, epigenetic changes, and telomere shortening^8–10^. Thus, obesity dramatically affects reproductive performance, particularly compromising oocyte quality.

Leptin is a main adipokine and its concentration rapidly increases in serum and follicular fluid of obese women^11^. Leptin is mostly known for its regulatory role on food intake and satiety^12,13^. At the ovarian level, leptin is known to control primordial follicle activation^14,15^, follicular growth and ovulation ^16^. Studies in mice lacking leptin (*ob/ob*) reported major perturbations in folliculogenesis, and prevalence of atretic follicles in the ovaries^17^. We have recently characterised the establishment of leptin resistance in the ovaries of diet-induced obese (DIO) mice^18^. The initial hyperactivation of leptin signalling after 4 weeks (wk) of high-fat diet (HFD) treatment contrasted with the failure in the activation of the isoform b of leptin receptor (ObRb) and the establishment of leptin resistance after 16 wk of HFD, a process mostly mediated by increased protein levels of the signalling inhibitor suppressor of cytokine signalling 3 (SOCS3)^18^. Therefore, obesity leads to dramatic changes in ovarian leptin signalling, with repercussions for folliculogenesis and ovarian function in general.

Mice lacking ObRb (*db/db*) and *ob/ob* mice have been largely used to study obesity and metabolic disorders^19,20^. Impaired leptin signalling in both models was shown to result in severe obesity, hyperphagia and compromised glucose metabolism^21,22^. Whereas *ob/ob* mice lack leptin production, *db/db* mice present systemic hyperleptinaemia in association with ObRb deficiency, which might result in noncanonical leptin signalling activation (ObRb-signalling independent). Importantly, the use of such models will elucidate how the dysregulation of different components of leptin signalling in the ovaries of obese mice might contribute to compromised folliculogenesis and oocyte quality^23,24^.

NOD-, LRR- and pyrin domain-containing protein 3 (NLRP3) is a large intracellular protein complex known to detect a broad range of endogenous (danger-associated molecular patterns, DAMPs) and environmental (pathogen-associated molecular patterns, PAMPs) signals, resulting in the formation and activation of the NLRP3 inflammasome. Apoptosis-associated speck-like protein containing a C-terminal caspase recruitment domain (ASC) is known to promote the interaction of NLRP3 with caspase-1 (CASP1) through an amino-terminal pyrin domain (PYD) and CARD domain^25^. Canonical NLRP3 inflammasome activation leads to the upregulation of pro-interleukin 1β (IL-1β) gene, through CASP1 activation and the release of the active proinflammatory cytokines IL-1β and IL-18^26,27^. Importantly, obesity has been associated with NLRP3 inflammasome activation^28–30^. Recent findings also revealed an important role of the NLRP3 inflammasome in follicular development in mice^31^. Hence, our recent work demonstrated the association between levels of ovarian leptin signalling and NLRP3 inflammasome activation in obese mice^32^. Nonetheless, the exact molecular mechanisms linking leptin signalling with NLRP3 inflammasome activation in the ovaries of obese mice, as well as the role of the aforementioned crosstalk on the modulation of inflammatory response, are yet to be fully understood.

Macrophages are key mediators of ovarian function^33^ and are known to present two main functional subtypes: the classically activated macrophages (M1), and the alternatively activated macrophages (M2). The M1 macrophages were shown to control folliculogenesis, ovulation and luteinisation^34^, whereas the M2 macrophages have been associated with vasculogenesis^35^, as well as profibrotic activity in advanced stages of ovarian cancer^34,36^. Importantly, previous studies have shown the macrophage infiltration of ovaries form obese mice^37^. Therefore, macrophage infiltration in the ovaries of obese mice might contribute to the pathophysiology of ovarian failure^38^. Importantly, the treatment of M1 human monocytes and macrophages with lipopolysaccharide (LPS) significantly induced the expression of NLRP3 and CASP1^39^. Also, recent studies have revealed that leptin activates the NLRP3 inflammasome and promotes M1 polarisation via the NLRP3 inflammasome in vitro^40^. Thus, evidence shows the involvement of leptin not only on macrophage polarisation but also in NLRP3 inflammasome activation, highlighting the importance of the crosstalk between leptin signalling, NLRP3 inflammasome activation and macrophage infiltration for the pathophysiology of ovarian failure in obese mothers.

In the present work, we hypothesise that temporal fluctuations in ovarian leptin signalling throughout obesity affect the inflammatory response in the organ, through the modulation of NLRP3 inflammasome activity and macrophage infiltration. Furthermore, we used genetically obese mice, to interrogate whether transcriptional changes in GCs associated with distinctive perturbations of leptin signalling, affect the expression of genes regulating NLRP3 inflammasome and macrophage infiltration, in this particular cellular compartment. Thus, we firstly analysed and characterised the transcriptome of GCs collected from *db/db* and *ob/ob* mice. Subsequently, we characterised the expression of NLRP3 inflammasome components in whole ovary extracts from *db/db* mice, followed by the comparative analysis of M1 macrophage marker genes in ovarian extracts from mouse models for DIO, pharmacological hyperleptinemia, and the genetically obese *db/db* and *ob/ob*.

## Materials and methods

### Animals

The animal protocols were performed in the Animal Facility at the Institute of Animal Reproduction and Food Research, Polish Academy of Sciences in Olsztyn. Mice were housed at standard temperature (23 °C) and were maintained in ventilated rooms under a standard photoperiod (12-h L:12-h D cycle) and humidity (50 ± 10%) with unlimited access to food and water. The mice were sacrificed by cervical dislocation. All animal studies were approved by and performed in accordance with the guidelines of the Local Animal Care and Use Committee for the University of Warmia and Mazury, Olsztyn (Agreement no. 80/2015, 38/2018, 74/2018, 19/2020). Guidelines for animal experiments followed EU Directive 2010/63/EU. Furthermore, the study is reported in accordance with ARRIVE guidelines. Breeding pairs of C57BL/6J (B6), B6.BKS(D)-Leprdb/J (*db/db*) and B6.Cg-Lepob/J (*ob/ob*) mouse strains were purchased from Jackson Laboratories (Bar Harbor, ME). Offspring mice were weaned at 21 days and housed in groups of 3–5 in plastic cages with fresh and clean sawdust bedding. Animal welfare was monitored during all experiments. At 8 weeks of age, female B6 mice from the DIO group were randomly assigned to the chow diet (CD) group fed a diet containing 13% of calories provided by fat (#5053, Picolab Rodent diet 20) or the HFD group fed a diet providing 59% of calories from fat (AIN-76A, 33% hydrogenated coconut oil; LabDiet). Animals were maintained on the diet for 4 or 16 wk (n = 7/group). Female homozygous *db/db* mice functionally deficient for the long form leptin receptor (n = 6/group) and *ob/ob* mice functionally deficient for leptin (n = 6/group) were kept on the CD until 12 wk of age. Lean littermates of the mutants served as controls (CT). In the pharmacological hyperleptinaemia model (LEPT; n = 7/group), female B6 mice at 8 wk of age were intraperitoneally administered either saline (SAL) or leptin (Recombinant Mouse Leptin, GFM26, Cell Guidance Systems) twice a day at a total dosage of 100 µg (injected at 09:00 and 21:00).

### Nuclear magnetic resonance

**Figure 1:**
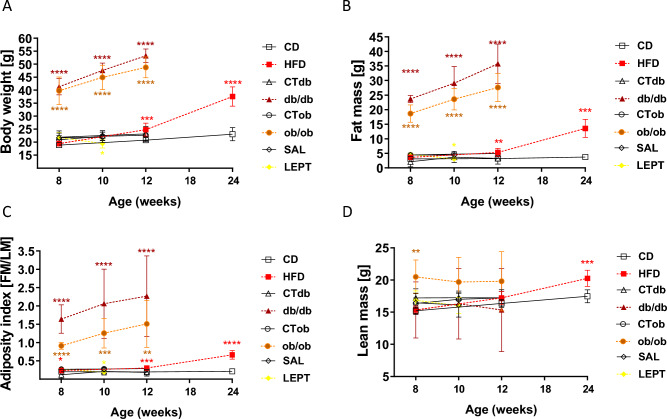
Phenotype characterisation of mouse models for obesity and pharmacological hyperleptinemia. Changes in (**A**) body weight, (**B**) fat mass, (**C**) adiposity index, and (**D**) lean mass measured by Time-Domain Nuclear Magnetic Resonance (TD-NMR spectroscopy; Bruker Minispec LF90 II) in the following models: (i) diet-induced obesity (DIO) protocol with mice fed chow diet (CD) or high fat diet (HFD) for 16 weeks; (ii) *db* model: mice lacking long isoform of leptin receptor (ObRb; db/db); (iii) *ob*model: mice lacking leptin (ob/ob); (iv) pharmacological hyperleptinemia model (LEPT) with mice injected either with saline (SAL) or 100 μg of leptin (LEPT) for 16 days. Lean littermates of the mutants served as controls (CT). Measurement was performed at 8, 12, 24 wk of age for DIO; 8, 10, 12 wk of age for *db/db* and *ob/ob* models: 8, 10 wk of age for LEPT. Different symbols represent various models: DIO (square), *db/db* (triangle), *ob/ob* (circle), LEPT (diamond). Colored lines correspond to experimental groups: HFD treatment (red), *db/db* model (brown), *ob/ob* model (orange), LEPT (yellow). Black lines correspond to control groups. Statistical differences were tested using Mann–Whitney test. Asterisks indicate significant differences *p < 0.05; **p < 0.01; ***p < 0.001; ****p < 0.0001.

The body composition of the mice was analysed using a Bruker Minispec LF90 II (Bruker, Rheinstetten, Germany). The nuclear magnetic resonance (NMR) method is based on measurements with high contrast between tissues (fat, body free fluid, muscle)^41^. Measurements of the body weight (BW), fat mass (FM), lean mass (LM) and adiposity index (AI, fat mass/lean mass) were performed at 8, 12 and 24 wk of age for DIO mice; 8, 10, and 12 wk of age for *db/db* and *ob/ob* mouse models; and 8 and 10 wk of age for LEPT mice (Fig. 1A–D). For the measurements, the mice were inserted into plastic tubes, immobilised by a tight-fitting plunger and scanned for approximately 2 min.

### Granulosa cell collection

Mouse ovaries were punctured with an insulin needle in M2 medium (M7167, Sigma–Aldrich) to release granulosa cell‒oocyte complexes from the antral follicles. Germinal vesicle stage oocytes were collected and denuded using size-specific glass needles and mouth pipettes. The released granulosa cells were collected into a tube, centrifuged (5 min, 600×*g*, 4 °C), washed with phosphate buffered saline (PBS) solution followed by centrifugation (5 min, 600×*g*, 4 °C) and resuspended in RLT buffer (1053393, Qiagen). The material was immediately frozen and stored at −80 °C until library preparation.

### Tissue collection

Animals were sacrificed, and genitalia were collected. After gonadal fat removal and quick rinsing in PBS, ovaries were separated and placed in tubes filled with TRI Reagent (T9424, Sigma–Aldrich) or radioimmunoprecipitation assay (RIPA) buffer for further mRNA or protein isolation. Material was stored at −80 °C until the time of analysis.

### Haematoxylin and eosin (HE) staining

For paraffin sections, slides were incubated at 65 °C for 20 min to melt paraffin, followed by dewaxing in the first xylene solution (520860119, POCH) for 20 min and the second xylene solution for 5 min. Next, slides were rehydrated through incubation in a series of alcohols: absolute alcohol (396420113, POCH), 90% ethanol (EtOH) and 70% ethanol for 5 min each. After air drying, slides were stained with Mayer’s Haematoxylin solution (MHS16, Sigma–Aldrich) for 3 min in a Coplin jar, followed by rinsing in cool running water for 2 min. Then, the slides were stained with eosin water-based solution (HT110216, Sigma–Aldrich) for 1 min and dipped in running water until eosin stopped streaking. The slides were then dipped in 70% EtOH, 90% EtOH, absolute alcohol and xylene and finally mounted using DPX mounting medium (06522, Sigma–Aldrich). The next day, images were captured using an Axio Observer Systems Z1 microscope (Carl Zeiss Microscopy GmbH, Hannover, Germany) and analysed using Zeiss ZEN 2.5 lite Microscope Software (Carl Zeiss, Germany).

### Immunofluorescent staining

In order to study the histological distribution of the macrophage marker cluster for differentiation 68 (CD68)^42^, immunofluorescent staining (IF) was performed in ovarian samples from mice treated with CD and HFD. Ovaries were fixed in 4% neutral buffered formalin (NBF, 432173427, Poch, Gliwice, Poland) at 4 °C for 24 h, and subsequently dehydrated in ethanol. Next, paraffin embedded ovarian tissues were sectioned into 5 μm slices. Cuts were then heated to remove paraffin (59 ℃, 120 min) and immersed in xylene and serious of ethanol solutions to rehydrate the tissue. Subsequently, tissues were permeabilised in 0.3% Triton X-100 (T8787, Sigma Aldrich) and heated in citrate buffer (10 mM, pH 6.0, 90 ℃, 40 min) for antigen retrieval. Tissues were maintained in blocking solution (2% BSA in PBS with 0.1% Tween20, PBST) for 2 h at RT followed by incubation in primary monoclonal anti-CD68 antibody (1:100, #14-0681-82, Thermo Fisher) overnight at 4 °C. The negative control sections were incubated with anti-immunoglobulin G (IgG, #A-11077, Thermo Fisher). On the next day slides were washed in PBST, followed by incubation with Alexa Fluor 594 (1:200, A21209, Thermo Fisher), and a series of washes in PBST. Finally, slides were covered with a drop of Prolong Gold medium with diamidino-2-phenylindole (DAPI) and sealed under cover slips. Images were captured using 40×/1.2A or 63×/1.4A oil immersion objectives on a LSM800 confocal microscope (Carl Zeiss, Germany).

### RNA isolation and cDNA synthesis

For mRNA extraction, ovaries were collected and placed in TRI reagent (T9424; Sigma–Aldrich) following the manufacturer’s instructions. Tissue was disrupted with a lancet, pipetted and incubated for 5 min at room temperature (RT). After centrifugation (9400×*g*, 15 min, 4 °C), the supernatants were collected and placed in fresh tubes, mixed with 100 µl of 1-bromo-3-chloropropane (BCP, BP151, Molecular Research Centre), and incubated for 10 min at RT. After centrifugation (13,500×*g*, 15 min, 4 °C), the aqueous phase was transferred to a fresh tube without disturbing the interphase and mixed with an equivalent volume of isopropanol. Samples were incubated for 1 h at −80 °C and centrifuged (20,000×*g*, 15 min, 4 °C). Subsequently, the samples were washed 3 times with 1 ml of 75% ethanol and incubated overnight at −80 ℃. The following day, samples were again centrifuged (20,000×*g*, 15 min, 4 °C), and after removing the supernatant, the RNA pellet was air-dried for 10 min and dissolved in 20 µl of RNase-free water (W4502, Sigma–Aldrich). RNA samples were stored at −80 ℃. The RNA quality and concentration were assessed with a NanoDrop. The ratio of the absorbance at wavelengths of 260 nm and 280 nm (A260/280) was analysed to confirm RNA quality and purity. A total of 2 µg of RNA was reverse transcribed using the Maxima First Strand cDNA Synthesis Kit for Real-Time Polymerase Chain Reaction (PCR) (K1642, Thermo Scientific) following the manufacturer’s instructions. The cDNA was then stored at − 20 °C for subsequent analysis.

### Real-time polymerase chain reaction

Real-time PCR relative analysis was performed in a 7900 Real-time System (Applied Biosystems) using a default thermocycler program for all genes: a 10-min preincubation at 95 °C was followed by 45 cycles of incubation for 15 s at 95 °C and 1 min at 60 °C, followed by a dissociation step (15 s at 95 °C, 15 s at 60 °C, and 15 s at 95 °C) to ensure single product amplification. Ribosomal protein L37 (*Rpl37*) was used as a housekeeping gene to normalise the results. Both the target gene and the housekeeping gene were run simultaneously in each run. The primers for *Nlrp3*, *Casp1, Il-1β, Il-18,* Adhesion G Protein-Coupled Receptor E1 (*Adgre1*)*,* Cd80 antigen (*Cd80*)*,* and Mannose receptor, C type 1 (*Mrc1*) were designed using Primer Express 3.0 software (Applied Biosystems) based on gene sequences in GenBank (National Centre for Biotechnology Information, sequences presented in Table 1). The primers were synthesised by Sigma–Aldrich. All reactions were carried out in duplicate in 384-well plates (4309849; Applied Biosystems) in a total solution volume of 12 µl ^43^. Relative mRNA quantification data were analysed with the Real-time PCR Miner algorithm^44^.

**Table 1:** Specific primers used for quantitative real-time PCR.

Gene name	Gene symbol	GenBank accession no	Sequences 5′–3′	Length (base pairs)
Adhesion G protein-coupled receptor E1	*Adgre1 *(*F4/80*)	NM_010130.4	F: TCCTGCTGTGTCGTGCTGTT R: TGTAGGAATCCCGCAATGATG	148
CD80 antigen	*Cd80*	NM_009855.2	F: ACGACTCGCAACCACACCAT R: CTGCCCCAAAGAGCACAAGT	130
Mannose receptor, C type 1	*Mrc1 *(*Cd206*)	NM_008625.2	F: TGGCTTGGGCTACAGGAGAA R: CTGGTGTCGTGGGTGTGGTA	177
NLR family pyrin domain containing 3	*Nlrp3*	NM_145827.4	F: TGGATGGGTTTGCTGGGATA R: TGCTTGGATGCTCCTTGACC	190
Caspase 1	*Casp1*	NM_009807.2	F: CATGCCGTGGAGAGAAACAA R: GGTGTTGAAGAGCAGAAAGCAA	151
Interleukin-1β	*Il-1β*	NM_008361.4	F: TTGACGGACCCCAAAAGATG R: GCTTCTCCACAGCCACAATGA	144
Interleukin-18	*Il-18*	NM_008360.2	F: GAAGAAAATGGAGACCTGGAATCA R: TCTGGGGTTCACTGGCACTT	157
Ribosomal protein L37	*Rpl37*	NM_026069.3	F:CTGGTCGGATGAGGCACCTA R: AAGAACTGGATGCTGCGACA	108

### Western blotting

The protein expression of NLRP3 was assessed by western blotting (n = 4–5/group). Ovaries were collected in RIPA supplemented with inhibitors and mechanically disrupted with a lancet. Then, lysates were incubated for 1 h on ice, mixing every 15 min. Subsequently, samples were centrifuged (20,000*g*, 4 ℃, 15 min) and the supernatant was collected and stored at −80 °C until the analysis was performed. The protein concentration was assessed using bicinchoninic acid assay (BCA, BCA1-1KT, Sigma Aldrich). A total of 20 μg of protein was loaded on 12% acrylamide gel, and after electrophoresis proteins were transferred to polyvinylidene difluoride (PVDF) membrane. Membranes were sectioned longitudinally in order to incubate each section with target and protein normaliser simultaneously. Next, membranes were blocked in 5% BSA (A2153, Sigma Aldrich) solution in PBST and incubated with mouse monoclonal anti- NLRP3 (1:1000, AG-20B-0014-C100, Adipogen), CASP1 (1:1000 ab108362, Abcam) and IL-18 (1:250 ab71495, Abcam) antibody solution overnight at 4 °C. Proteins were detected after incubation of the membranes with anti-mouse alkaline phosphatase-conjugated antibody (1:20,000, 31321, Thermo Scientific) for 2 h at RT. Immune complexes were visualised using the alkaline phosphatase or by enhanced chemiluminescence (ECL) substrate visualisation. Blots were scanned using the Molecular Imager VersaDoc MP 4000 System (BioRad, Hercules, California, USA) and specific bands quantified with ImageLab Software (BioRad). The results were normalised with β-actin (1:10,000, A2228, Sigma-Aldrich) or glyceraldehyde 3-phosphate dehydrogenase (GAPDH, 1:2500, ab9485, Abcam) run in the same membrane, as target protein.

### RNA-seq library generation

GCs were collected in tubes with RLT buffer (1053393, Qiagen) and stored at −80 °C for further analysis. Afterwards, RNA sequencing (RNA-seq) libraries were generated as previously described, with minor changes. Magnetic beads with pre-annealed Smart-seq2 oligo-dT sequences (MyOne C1, Invitrogen) were used to capture mRNA. The beads were then diluted in 10 μl of reverse transcriptase mix (100 U, SuperScript II, Invitrogen; 10 U, RNAsin, Promega), 1× Superscript II First-Strand Buffer, 2.5 mM dithiothreitol (DTT, Invitrogen), 1 M betaine (Sigma–Aldrich), 9 mM magnesium chloride (MgCl2, Invitrogen), 1 μM Template-Switching Oligo (Exiqon), and 1 mM deoxyribonucleotide triphosphate (dNTP) mix (Roche) and incubated for 60 min at 42 °C, followed by 30 min at 50 °C and 10 min at 60 °C^45,46^. The amplification of the cDNA was performed after adding 11 μl of 2 × KAPA HiFi HotStart ReadyMix and 1 μl of 2 μM ISPCR primer (insert—Smart 2013, Full-length RNA-seq 2014) and incubating at 98 °C for 3 min, followed by nine cycles of 98 °C for 15 s, 67 °C for 20 s, 72 °C for 6 min and finally 72 °C for 5 min. cDNA was purified with AMPure beads (Beckman Coulter) and eluted into 20 μl of nuclease-free water (P1195; Promega). All libraries were prepared from 100 to 200 pg of cDNA using the Nextera XT Kit (Illumina) following the manufacturer’s instructions. The final cDNA libraries were pooled and sequenced on an Illumina NextSeq500 instrument in 75-base-pair (bp) single-read high output mode at the Babraham Institute Sequencing Facility.

### Identification of differentially expressed genes

The quality of raw reads was evaluated by means of FASTQC (https://www.bioinformatics.babraham.ac.uk/projects/fastqc/). Low-quality reads and adapters were removed using cutadapt software (version 1.18)^47^. Then, reads were mapped to the mouse reference genome (GRCm39) using STAR software (version 2.7.10a)^48^. Afterwards, raw counts per gene were calculated using the feature Counts tool (version 2.0.1)^49^. The differentially expressed genes (DEGs) and corresponding P-adjusted values were determined by means of R statistical software (version 4.1.3) using the DESeq2 package (version 1.34.0)^50^. The threshold for significantly different expression was set at P-adjusted ≤ 0.05. Visual presentation of the results was performed in R software using the ggplot2 (version 3.3.6)^51^, Venn Diagram (version 1.7.3) and pheatmap (version 3.1.1)^52^ packages.

### Functional enrichment analysis

Functional analysis of the DEGs was performed based on the Gene Ontology (GO) database using the clusterProfiler (version 4.2.2)^53^, DOSE (version 3.20.1)^54^, biomaRt (version 2.50.3)^55^ and org.Mm.eg.db (version 3.14.0)^56^) packages of R software, with the established criterion P-adjusted ≤ 0.05. The visual presentation of the results was prepared using R software with ggplot2.

### Statistical analysis and data representation

Data were analysed using GraphPad Prism Software (Version 9.01, GraphPad Software, Inc.; La Jolla, CA, USA). One-way analysis of variance (ANOVA) was conducted when more than two groups were compared. The D’Agostino-Pearson omnibus normality test was performed followed by the nonparametric Mann‒Whitney test or multiple unpaired t tests with statistical significance determined using the Bonferroni-Sidak method, depending on the experiment. The statistical analysis of each experiment is presented in the figure legends. The error bars represent the standard error of the mean. Significance was defined as values of p < 0.05.

## Results

### Genetic and diet induced obesity, but not pharmacological hyperleptinemia, are associated with increased body weight and fat mass

To characterise the role of leptin in the modulation of NLRP3 inflammasome profile changes and subsequent macrophage activation in the ovary during obesity, we used two genetically obese *db/db* and *ob/ob* mice, DIO mice, and the leptin treated LEPT mice. The phenotype measurements were taken between 8 and 24 wks of age in comparison to the respective controls. The starting BWs of *db/db* and *ob/ob* mutants were approximately 40 g (g), whereas the remaining mice were approximately 20 g (Fig. 1A). As expected, both *db/db and ob/ob* mice were considerably fatter at 12 wk of age, with an average BW of 53 g for *db/db* mice and 49 g for *ob/ob* mice (Fig. 1A, p < 0.0001), whereas the HFD group weighted on average 33 g at 24 wk of age (Fig. 1A, p < 0.0001). Concerning the LEPT mice, BW decreased from 20 to 17 g on average between 8 and 10 wk of age (Fig. 1A, p < 0.05). The FM in *db/db* and *ob/ob* mice increased from 8 to 12 wk of age by approximately 11 g in both models, reaching a total of 32 g in *db/db* mice and 23 g in *ob/ob* mice greater than that in the control mice (Fig. 1B, p < 0.0001). The FM of the HFD group was significantly higher at 12 wk of age and was 9 g greater at 24 wk (Fig. 1B, p < 0.01 and p < 0.001 respectively), whereas in LEPT mice, FM was lower at 10 wk of age (Fig. 1B, p < 0.05). The AI in *db/db* and *ob/ob* mice increased from 8 to 12 wk of age, with a gain of approximately 0.5% in AI in both models that was 0.94% greater in *db/db* and 0.16% in *ob/ob* mice compared to that in the control mice (Fig. 1C, p < 0.0001 and p < 0.01 respectively). The HFD group presented a 1.17% increase in AI (Fig. 1C, p < 0.0001), and LEPT mice showed a decrease of 0.06% in AI compared to that of the control group (Fig. 1C, p < 0.05). The LM in *db/db* mice was lower by 2 g, while the LM in *ob/ob* mice was 4 g higher (Fig. 1D, p < 0.01) at the start of the protocol. In the HFD group, a gain in LM was observed between 8 and 24 wk of age, with a final gain of 3 g, compared to that in the controls (Fig. 1D, p < 0.001). Thus, our genetically obese *db/db* and *ob/ob* mice, as well as the DIO mice, presented an obese phenotype in contrast with the LEPT mice, which maintained a lean phenotype throughout the experimental protocol. All controls (CT) also maintained a lean phenotype as expected.

### Transcriptional differences in in granulosa cells from obese db/db and ob/ob mice do not reflect phenotypic similarities

**Figure 2:**
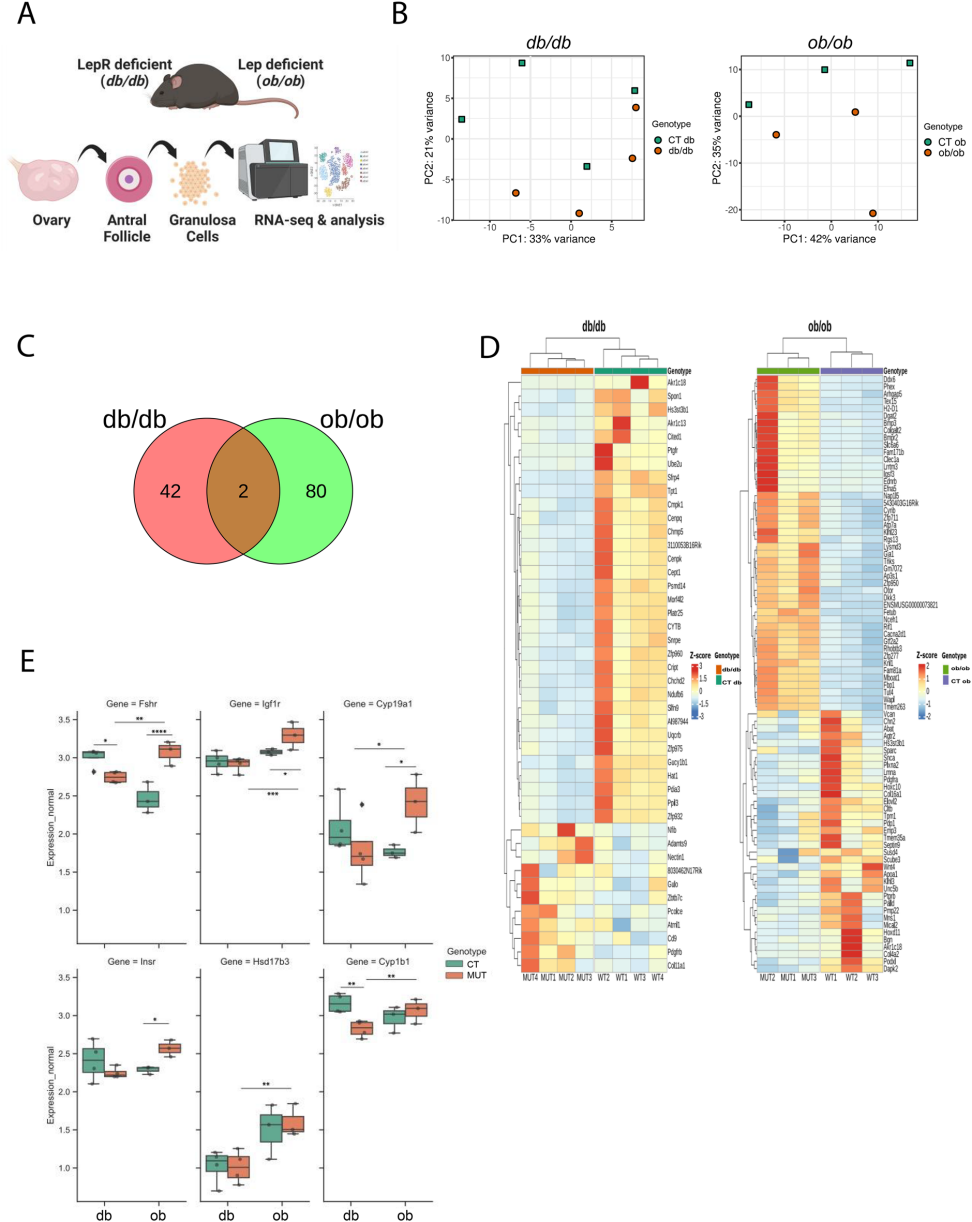
Transcriptome analysis of granulosa cells collected from genetically obese mice: lacking long isoform of leptin receptor (*db/db*) or leptin (*ob/ob*). (**A**) Experimental design: granulosa cells (GCs) collected from antral follicles isolated from ovaries from mice lacking long isoform of leptin receptor (*db/db*) and mice lacking leptin (*ob/ob*) were subjected to RNA sequencing (RNA-seq) analysis. (**B**) Principal Component Analysis (PCA) presenting the GCs from *db/db* and *ob/ob* mice showing no definite sample clustering according to the global transcriptome profile. Controls are depicted in green while mutants in red for both strains. (**C**) Venn diagram depicts differentially expressed genes (DEGs) identified in GCs collected from *db/db* (indicated in red) and *ob/ob* (indicated in green) mice. The overlap represents DEGs that are common for both groups. Only two common DEGs indicate a distinct genetic profile between the mouse strains. (**D**) Heatmaps showing unsupervised hierarchical clustering of the identified DEGs (p-adjusted ≤ 0.05) in GCs from *db/db* and *ob/ob* mice. The color scale of the heatmaps indicate expression level where red blocks represent up- and blue blocks down-regulated genes. Samples from mutant db/db mice are indicated in red while its control (CT db)in green. Mutant ob/ob are highlighted as lighter green, while controls (CT ob) in violet. (**E**) Box-whisker plots illustrating the expression of genes in GCs collected from *db/db* and *ob/ob* mice after RNA-seq analysis. Selected genes associated with steroidogenesis were studied: *Follicle stimulating hormone receptor* (*Fshr*),* Insulin-like growth factor I receptor* (*Igf1r*), *Cytochrome P450, family 19, subfamily A1 *(*Cyp19a1*), *Insulin receptor substrate 1* (*Insr1*),* Hydroxysteroid 17-beta dehydrogenase 3* (*Hsd17b3*) and *Cytochrome P450 family 1 subfamily B member 1* (*Cyp1b1*). *CT* (*CT db or CT ob*)*-lean controls for mutants*; MUT-mutant mice (db/db or ob/ob). Asterisks indicate significant differences (∗p < 0.05; ∗∗p < 0.01; ****p < 0.0001). Expression level presented as arbitrary units (AU).

We first analysed the transcriptome of GCs collected from antral follicles isolated from *db/db* and *ob/ob* mice (Fig. 2A). RNA-seq provided 92.77 million raw reads, ranging from 3.59 million to 12.98 million reads per sample. After removing adapters and low-quality reads (Phread score < 20), the remaining 92.66 million high-quality reads (3.58 to 12.96 million per sample) were mapped to the mouse reference genome (GRCm39) with the number of aligned reads ranging from 3.41 to 12.45 million per sample. An average of 87.5% of reads were mapped to unique locations. The total number of genes expressed in mouse GCs ranged from 17,934 to 23,270 (Supplementary Fig. 1A, Table S1). Principal component analysis (PCA) revealed slight differences in the gene expression profile in GCs in the presented models, with no defined clustering pattern (Fig. 2B). Volcano plots presented significant changes (p-adjusted ≤ 0.05) in the gene expression profile of GCs from the controls (CT) compared to mutant (MUT; db/db and ob/ob) mice in both mouse strains (Supplementary Fig. 1B). A total of 44 genes were differentially expressed genes (DEGs) in the *db/db* model, with 11 up- and 33 downregulated genes (Table S2). In the *ob/ob* model, we found 82 DEGs, with 46 up- and 36 downregulated genes (Table S3). Among the DEGs from both models, only two genes, namely, *aldo–keto reductase family 1, member C18* (*Akr1c18*) and *heparan sulfate* [*glucosamine*]* 3-O-sulfotransferase* (*Hs3st3b1*), overlapped and were downregulated in both mutants (Fig. 2C, D). Both models present dramatic changes in metabolism and are infertile, as loss-of-function studies of *Akr1c18* showed abnormalities in the oestrous cycle ^57^. Conversely, the *Hs3st3b1* gene is known to be involved in carbohydrate and protein metabolic processes ^58^. The heatmap shows the expression profile of DEGs across samples from each group and mouse model (Fig. 2D). To investigate the nature of the DEGs, gene ontology (GO) analysis was performed. Genes were classified into three main categories: “biological process”, “cellular component” and “molecular function”. The gene expression profiles of GCs from the *ob/ob* and *db/db* mouse models showed clear differences, which might be linked to the different patterns of impaired leptin signalling. In the *db* model, 33 downregulated genes were associated with “steroid metabolic process” and “progesterone metabolic process” (Table S4). Hence, the genes *Akr1c18* and *Akr1c13*, which are known to be involved in progesterone metabolism in follicles ^59,60^, were downregulated in *db/db* mice compared to the control mice. Furthermore, the downregulation of *prostaglandin F2 alpha receptor* (*Ptgfr*) in *db/db* mice impaired follicular development, as prostaglandins are known to tightly control late follicular development ^61^ and ovulation ^62^. Concerning the upregulated genes in *db/db* mice, terms such as “cardiac muscle tissue development” presented high significance (Supplementary Fig. 1C), being associated with the genes *platelet-derived growth factor receptor beta* (*Pdgfrb*), *collagen type XI alpha 1* (*Col11a1*), and *A disintegrin-like and metallopeptidase reprolysin type with thrombospondin type 1 motif 9 *(*Adamts9*). Importantly, *Pdgfrb* is known to mediate the effects of platelet-derived growth factor BB (PDGF-BB), an oocyte-specific ligand responsible for the control of follicular development, angiogenesis, and vascular permeability ^63–65^. Furthermore, *Col11a1* and *Adamts9* are well-established regulators of extracellular matrix (ECM) remodelling during follicular development. With regard to the *ob* model, GO analysis of downregulated genes revealed highly significant terms such as “collagen-containing extracellular matrix”, comprising the genes *Col4a2* and *Col16a1* (Supplementary Fig. 1C), which are important components of the ECM necessary for follicular development and ovulation ^66^. Furthermore, developmental genes from the Hox family, such as *homeobox C10* (*Hoxc10*) and *Hoxd11*, known to regulate GC function, ovarian follicle maturation ^67^, were also downregulated. Regarding the *ob* model, the term “negative regulation of small molecule metabolic process” from GO analysis was associated particularly with the upregulated genes *diacylglycerol acyltransferase 2* (*Dgat2*) and *fructose-1,6-bisphosphatase 1* (*Fbp1*) (Table S7), which were previously associated with lipid metabolism in GCs ^68–70^. Finally, we tested a particular subset of genes regulating steroidogenesis in GCs and observed opposing trends in gene expression between the models (Fig. 2E). Specifically, the genes *follicle stimulating hormone receptor *(*Fshr*)*, insulin-like growth factor I receptor *(*Igf1r*)*, cytochrome P450, family 19, subfamily *(*Cyp19a1*)*, insulin receptor substrate 1 *(*Insr1*)*, hydroxysteroid 17-beta-dehydrogenase 3 *(*Hsd17b3*) and *cytochrome P450 family 1 subfamily B member 1 *(*Cyp1b1*) tended to be downregulated in *db/db* mice but upregulated in *ob/ob* mice compared to the controls (Fig. 2E, p < 0.05). Thus, in both genetically obese mice, follicular development is impaired, with metabolic dysregulation in the *ob/ob* mice resulting from the lack of leptin signalling, whereas in the *db/db* mice, noncanonical leptin action was associated with the upregulation of genes controlling vasculogenesis. Ultimately, noncanonical leptin activity in GCs from *db/db* mice leads to inhibition of steroidogenesis genes expression, in comparison with the *ob/ob* mice which lacks leptin signalling.

### Leptin signalling modulates the NLRP3 inflammasome profile and macrophage M1 polarisation in the ovaries of obese mice

**Figure 3:**
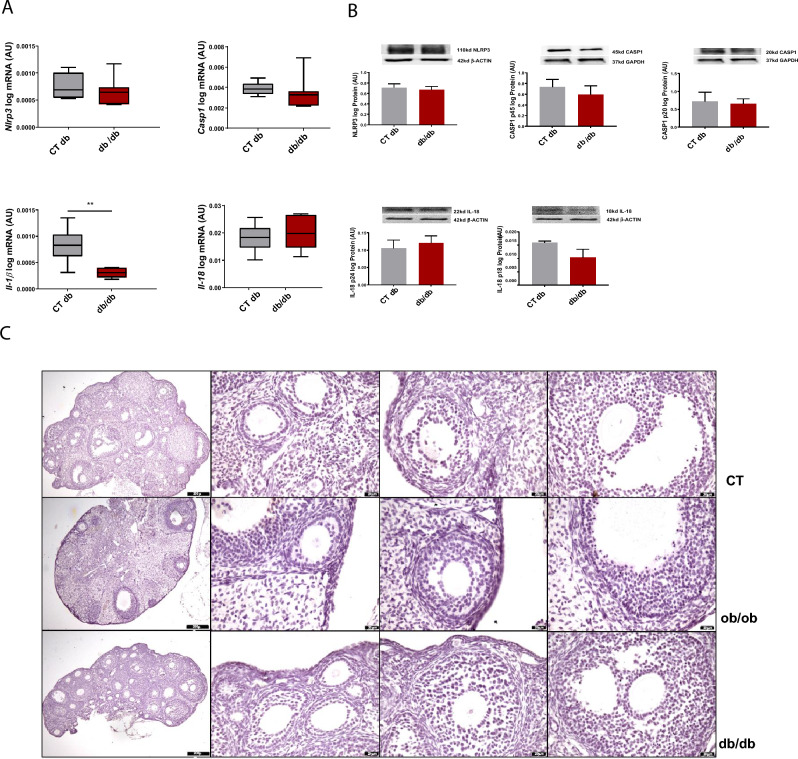
NOD-like receptor protein 3 (NLRP3) inflammasome activation persists in the ovaries of *db/db* homozygous mice. (**A**) Quantification of mRNA levels of NOD-like receptor protein 3 (*Nlrp3*), caspase-1 (*Casp1*), interleukin-1β (*Il-1β*), *Il-18* in mice lacking long isoform of leptin receptor (*db/db*) determined by real-time Polymerase Chain Reaction (PCR). (**B**) Abundance of NLRP3, pro CASP1 p45, CASP1 p20, pro IL-18 p24, and IL-18 p18 protein in *db/db* model measured by Western Blot analysis. Original full blots or sectioned membranes are presented in Supplementary Fig. 2. Data were normalised to ribosomal protein L37 (*Rpl37*) mRNA expression and β-actin or glyceraldehyde 3-phosphate dehydrogenase (GAPDH) protein expression. Bars represent mean ± SEM. Statistical analysis between groups was carried out using Mann–Whitney test. Number of samples: n = 6 for real-time PCR analysis and n = 4 for immunoblots. Asterisks indicate significant differences (∗∗p < 0.01). *AU* arbitrary units; CT (*db*)—lean control for db mutants. (**C**) Morphological changes in mice ovaries from *db/db* and mice lacking leptin (*ob*/*ob*) models. Upper panel: representative haematoxylin/eosin (H&E) staining of a normal mouse ovary (CT: control). Medium panel: representative H&E staining of *ob/ob* mouse ovary. Lower panel: representative H&E staining of *db/db* mouse ovary. Each panel shows the following sections: whole ovary, primary follicle, secondary follicle, antral follicle, presented from left respectively. Ovaries of both *db/db* and *ob/ob* mice contained more atretic follicles, smaller number of antral follicles and no corpora lutea in comparison with control mice. Scale bars represent 200 or 20 μm.

**Figure 4:**
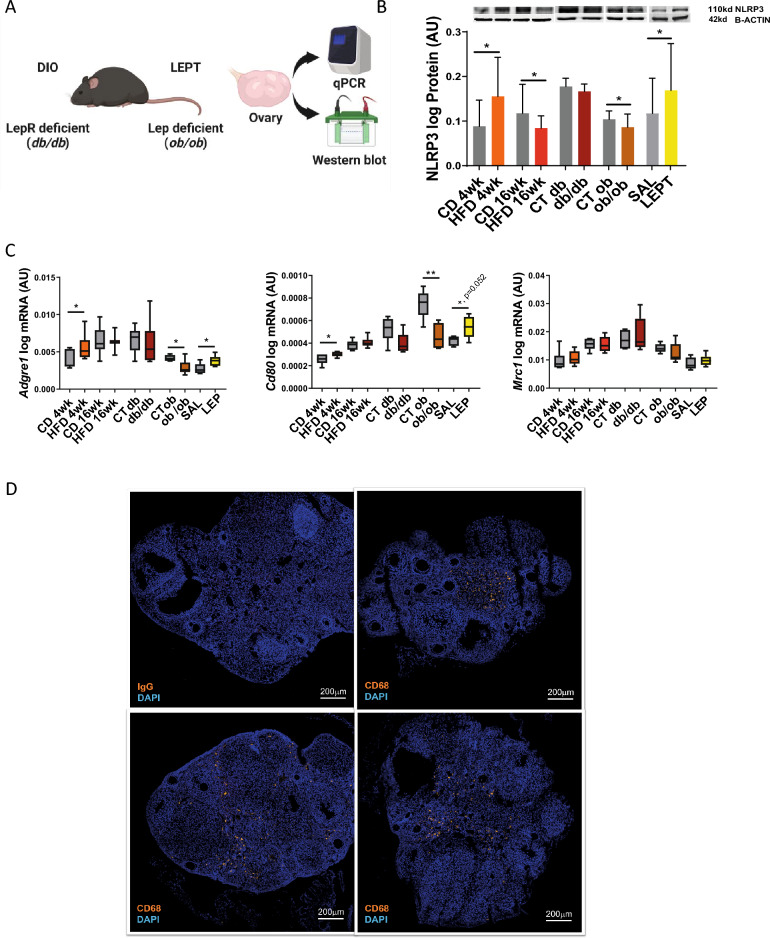
Leptin receptor isoform b—independent leptin signalling controls the expression of NOD-like receptor protein 3 (NLRP3) and M1 macrophage markers in the ovary of obese mice. (**A**) Experimental design: ovaries were collected from mice belonging to following different groups: (i) from diet-induced obesity (DIO) protocol where mice were fed either chow diet (CD) or high fat diet (HFD) for 4 or 16 weeks (wk); (ii) genetic obesity model, mice lacking long isoform of leptin receptor (*db*); (iii) and genetic obesity model, mice lacking leptin (*ob*); (iv) pharmacological hyperleptinemia model (LEPT), mice injected either with saline (SAL) or 100 μg of leptin (LEP) for 16 days and submitted to mRNA or protein level analysis via quantitative Polymerase Chain Reaction (qPCR) or Western Blot, respectively. (**B**) Abundance of NOD-like receptor protein 3 (NLRP3) protein in DIO, *db/db*, *ob/ob* LEPT models measured by Western Blot analysis; blots for DIO, *ob/ob*, LEPT models previously published ^32^. Original full blots or sectioned membranes are presented in Supplementary Fig. 3. For Fig. 3A, (**C**) quantification of mRNA levels of *Adhesion G Protein-Coupled Receptor E1* (*Adgre1*), *Cd80 antigen* (*Cd80*), *Mannose receptor, C type 1* (*Mrc1*) in DIO, *db/db*, *ob/ob*, LEPT models determined by real-time PCR. Data were normalised to ribosomal protein L37 (*Rpl37*) mRNA expression and β-actin protein expression. Bars represent mean ± SEM. Statistical analysis between groups was carried out using Mann–Whitney test. Number of samples: n = 6–7 for qPCR analysis and n = 4–8 for immunoblots. Asterisks indicate significant differences (∗p < 0.05; ∗∗p < 0.01; ^x^p = 0.052). AU- arbitrary units; *CT *(*db*)* and CT *(*ob*)*-lean controls for respective db and ob mutants* (D) Immunofluorescent localization of macrophage marker CD68 in ovarian sections of DIO mice. Upper left panel: representative staining of immunoglobulin G in DIO mice ovaries to confirm staining specificity. Three remaining panels stained with CD68 in orange, counterstaining with DAPI in blue. CD68 protein was localized in stromal compartment. Images are representatives of 3 biological replicates. Scale bar represents 200 μm.

In order to further characterise the role of leptin in the modulation of NLRP3 inflammasome profile changes and subsequent macrophage activation in the ovary during obesity, we started by characterising the expression of NLRP3 components in the ovaries of *db/db* mice. We observed no changes in the mRNA and protein levels of NLRP3, CASP1 and IL-18 in the ovaries of *db/db* mice, except for mRNA levels of *Il-1β* which were decreased in *db/db* (Fig. 3A, B, Supplementary Fig. S2). We also confirmed the presence of antral follicles in the ovaries of *db/db* after staining with haematoxylin and eosin (Fig. 3C). Subsequently, we used our previous results on NLRP3 inflammasome components at mRNA and protein levels from DIO, LEPT, and *ob/ob* mice ^32^ and reanalysed the data together with the *db/db* datasets (n = 4–8/group) (Fig. 4A). We observed that NLRP3 protein expression was upregulated after 4 wk of HFD feeding but downregulated after 16 wk of HFD feeding (Fig. 4B, p < 0.05, Supplementary Fig. S3A) or in ovaries from *ob/ob* mice (Fig. 4B, p < 0.05, Supplementary Fig. S3C). Moreover, the ovaries of LEPT mice presented increased protein levels of NLRP3 (Fig. 4B, p < 0.05, Supplementary figure S3D). Nonetheless, in the *db* model, no changes in NLRP3 levels were observed in the db/db mice (Fig. 4B, p  > 0.05, Supplementary Fig. S3B). Subsequently, we assessed the potential cross-talk between ovarian leptin signalling, NLRP3 inflammasome activation, and macrophage phenotype characterisation. We analysed the expression of the macrophage markers *Adgre1*, a pan-macrophage marker; *Cd80*, an M1 macrophage surface marker; and *Mrc1*, an M2 macrophage surface marker. Real-time PCR analysis (n = 6/group) revealed increased mRNA expression of *Adgre1* and *Cd80* after 4 wk of HFD compared to the control group (Fig. 4C, p < 0.05). Furthermore, the mRNA levels of *Adgre1* and *Cd80* were increased in the ovaries of the LEPT model (Fig. 4C, p < 0.05 and p = 0.052, respectively). The mRNA levels of *Adgre1* and *Cd80* were significantly decreased in the *ob/ob* mice compared to the controls (Fig. 4C, p < 0.05 and p < 0.01, respectively). No significant changes were observed in the *db/db* group for all chosen markers or for *Mrc1* mRNA levels in all analysed groups (Fig. 4C). Finally, we characterised the distribution of the macrophage marker CD68 ^42^ in the ovarian tissue by IF (n = 3, samples from CD and HFD treated mice randomly presented), and confirmed that macrophages were mostly visualised in the interstitial tissue surrounding the follicles (Fig. 4D, CD68 staining in orange; nuclear staining by DAPI in blue). No signal was obtained for IgG staining (Fig. 3D). In the present experiment, we confirmed that during obesity progression, ovarian leptin signalling regulates NLRP3 inflammasome activation and the expression of genes regulating M1 macrophage infiltration. In the later stages of obesity, failure in NLRP3 inflammasome activation and M1 macrophage regulation is associated with the establishment of ovarian leptin resistance, which is associated with the disruption of ObRb-dependent (canonical) and ObRb-independent (noncanonical) signalling.

### The association between leptin signalling, NLRP3 inflammasome activation and macrophage infiltration genes is not preserved in granulosa cell compartment

**Figure 5:**
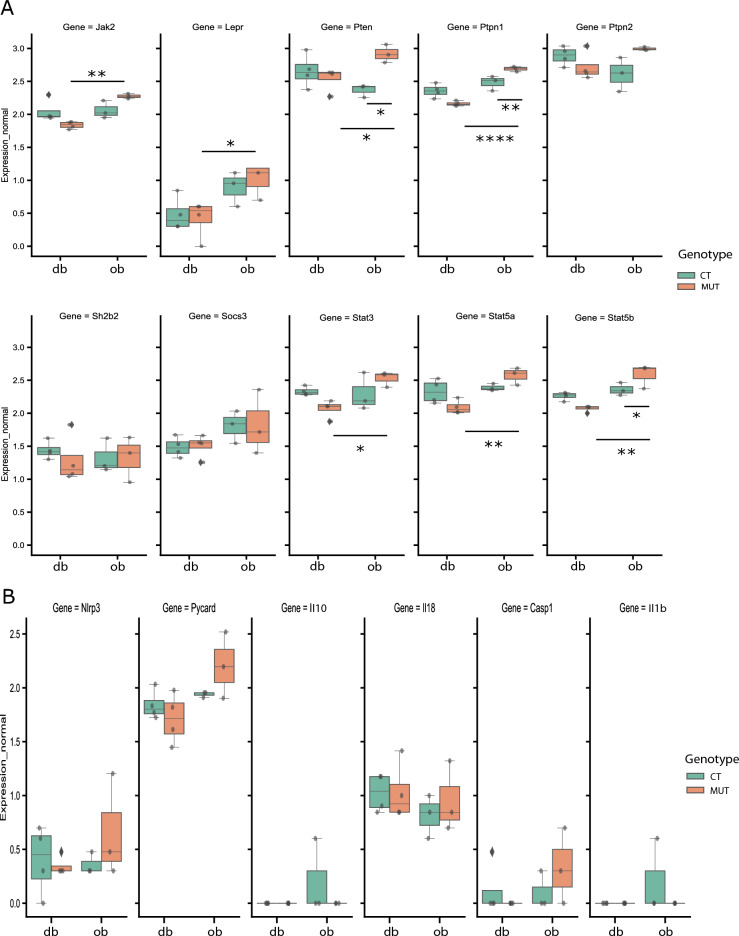
Expression of leptin signalling and NOD-like receptor protein 3 (NLRP3) inflammasome components in granulosa cells collected from genetically obese mice: lacking long isoform of leptin receptor (*db*) or leptin (*ob*). Box-whisker plots illustrating the expression of genes involved in leptin signalling (**A**) and components of NOD-like receptor protein 3 (NLRP3) inflammasome (**B**) in granulosa cells (GCs) collected from mice lacking long isoform of leptin (*db/db*) and mice lacking leptin (*ob/ob*) after RNA sequencing (RNA-seq) analysis. (**A**) *Janus kinase 2 *(*Jak2*)*, Leptin receptor *(*Lepr*)*, Phosphatase and tensin homolog *(*Pten*)*, Protein tyrosine phosphatase 1 *(*Ptpn1*)*, Ptpn2, SH2B adaptor protein 2 *(*Sh2b2*)*, Suppressor of cytokine signaling 3 *(*Socs3*)*, Signal transducer and activator of transcription 3 *(*Stat3*)*, Stat5a, Stat5b*. (B) *Nlrp3*, *PYD and CARD domain containing *(*Pycard*)*, Interleukin-10 *(*Il-10*)*, Il-18, Caspase-1 *(*Casp1*)*, Interleukin-1β* (*Il-1β*). *CT—control for mutants *(*CT db or CT ob*)*;* *MUT* mutant mice (db/db or ob/ob). Asterisks indicate significant differences (∗p < 0.05; ∗∗p < 0.01; ****p < 0.0001). Expression level presented as arbitrary units (AU).

**Figure 6:**
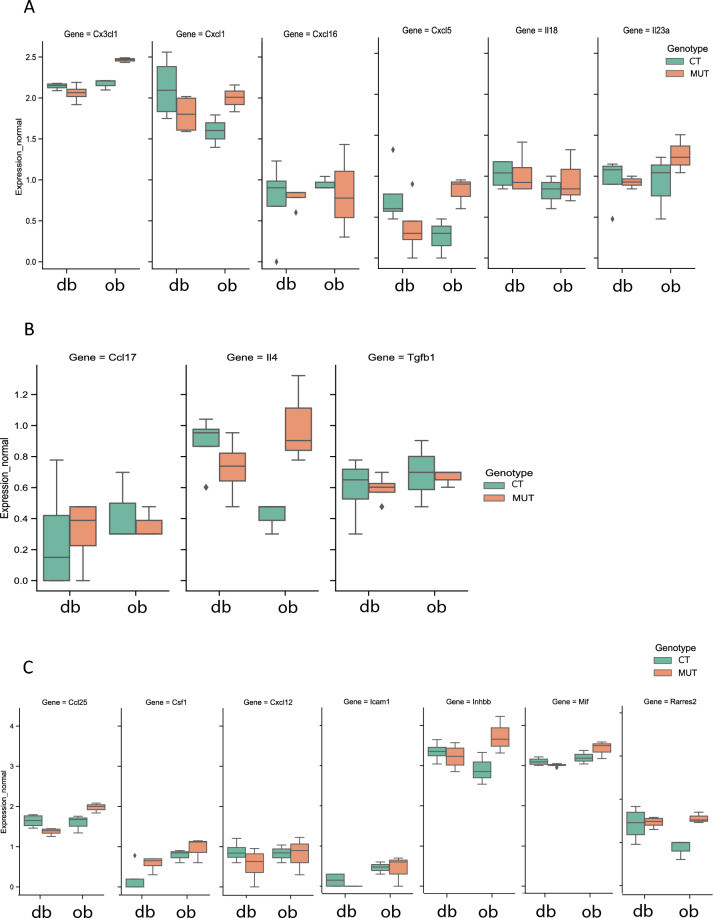
Inflammatory markers associated with macrophage polarization and infiltration in granulosa cells do not reflect differences in leptin signalling in mice lacking long isoform of leptin receptor (*db*) or leptin (*ob*). Box-whisker plots illustrating the gene expression of chemokines and cytokines secreted from granulosa cells (GCs) associated with macrophage commitment to M1 (**A**) and M2 (**B**), as well as macrophage infiltration (**C**) in the ovary of mice lacking long isoform of leptin (*db/db*) and mice lacking leptin (*ob/ob*) mice after RNA sequencing (RNA-seq) analysis. (**A**) *Chemokine *(*C-X3-C motif*)* ligand 1* (*Cx3cl1*), *chemokine *(*C-X-C motif*)* ligand 1* (*Cxcl1*), *Cxcl16, Cxcl5, interleukin 18* (*Il-18*), *Il-23a*. (**B**) *Chemokine *(*C–C motif*)* ligand 17* (*Ccl17*), *Il4*, *Transforming growth factor, beta 1* (*Tgfb1*). (**C**) *Ccl25, colony stimulating factor 1* (*Csf1*), *Cxcl12*, *intercellular adhesion molecule 1* (*Icam1*)*, inhibin beta-B* (*Inhbb*), *macrophage migration inhibitory factor* (*Mif*), *Retinoic acid receptor responder protein 2 *(*Rarres2*). *CT—control for mutants *(*CT db or CT ob*)*;* *MUT* mutant mice (db/db or ob/ob). Expression level presented as arbitrary units (AU).

Finally, we asked whether the association among leptin signalling, NLRP3 inflammasome activation, and mediation of M1 macrophage infiltration previously observed in the whole ovary was preserved in the GC compartment. After plotting leptin signalling components gene expression from our RNA-seq datasets, we observed that leptin signalling genes were consistently upregulated in GCs from *ob/ob* (Fig. 5A, p < 0.05), in comparison with the *db/db* mice but no changes were found between controls and mutants (Fig. 5A) or in NLRP3 inflammasome genes (Fig. 5B). No differences were found in the expression of genes regulating M1 (Fig. 6A), M2 (Fig. 6B) or general markers of macrophage infiltration (Fig. 6C). Generally, the present experiment revealed that in addition to the contrasting profiles of gene expression in GCs between *db/db* and *ob/ob* models, which were particularly evidenced by the discrepancies in steroidogenesis regulation, no differences were observed in the crosstalk between leptin signalling, the NLRP3 inflammasome and the regulation of M1 macrophage infiltration in the GC compartment.

## Discussion

The present report describes the link between changes in ovarian leptin signalling and the mounting inflammatory response in obese mice, particularly concerning the regulation of NLRP3 inflammasome activation and the mediation of M1 macrophage infiltration. Obese women show reduced fertility, often associated with ovarian failure characterised by altered folliculogenesis and decreased oocyte quality ^4,7^. Hence, the complexity of ovarian pathophysiology in obese mothers substantiates the importance of molecular studies characterising GC dysfunction during obesity. Therefore, we analysed the transcriptome of GCs collected from antral follicles isolated from genetically obese *db/db* and *ob/ob* mice to pinpoint the effects of ObRb (canonical) and non-ObRb (noncanonical)-mediated leptin signalling on GC function regulation during obesity. We observed that in the ovaries of obese mice, increased canonical and noncanonical leptin signalling regulates NLRP3 inflammasome activation and concomitant increased expression of genes mediating M1 macrophage infiltration. Furthermore, IF for the macrophage marker CD68 in ovarian samples from DIO mice and gene expression analysis of GCs collected from genetically obese mice revealed that the aforementioned relationship is seen exclusively in ovarian compartments other than the GC compartment.

We have recently characterised the link between the levels of ovarian leptin signalling and NLRP3 inflammasome activation in obese mice ^32^. Hence, with the establishment of leptin resistance in the ovaries after 16 wk of HFD treatment, the association between increased ovarian leptin signalling and activation of the NLRP3 inflammasome observed at 4 wk of HFD and after LEPT treatment was lost. The importance of active leptin signalling for NLRP3 inflammasome regulation in the ovaries during obesity progression was corroborated by the downregulation of NLRP3 protein in the ovaries of genetically obese *ob/ob* mice, a model known for its extreme obesity but absence of circulating leptin^71^. However, the extent to which leptin actions are mediated through the ObRb receptor (canonically) or through any other cellular receptor (noncanonically) remains unknown. We presently used the *db/db* mouse, known to have circulating leptin present but no expression of ObRb^72^, rendering any putative leptin signalling possible through receptors other than ObRb (noncanonically). Hence, NLRP3 protein levels were unchanged in the ovaries of *db/db* mice, suggesting that leptin can regulate the NLRP3 inflammasome through receptors other than ObRb (noncanonically). As a result, the disruption of ovarian leptin signalling during DIO possibly comprises not only ObRb (canonical) but also other receptors mediating leptin signalling (noncanonical). Thus, ovarian canonical and noncanonical leptin signalling control the activity of the NLRP3 inflammasome in the course of obesity in mice.

Macrophages are abundantly present in the ovary and exhibit different phenotypes and stages of activation, which are mostly modulated in response to endocrine changes throughout the oestrous cycle^33^. Presently, we sought to uncover the link between the changes in ovarian leptin signalling observed during obesity progression and the phenotypes of macrophages present locally. Previous studies have shown that the expression of the macrophage markers *F4/80* (*Adgre1*) and *Cd11c* (M1 macrophage markers) at the mRNA level was significantly increased in the ovaries of mice treated for 16 wk with HFD, whereas no changes were observed for *Cd206* (M2 macrophage marker)^73^. Furthermore, DIO and ageing have been associated with increased M1 macrophages in B6 mouse ovaries but without changes in M2 macrophages^74^. Presently, we have used the conserved marker genes *Adgre1* (pan-macrophage marker) and *Cd80,* which are expressed in proinflammatory macrophages^75^ (hereafter named M1 markers), and the gene *Mrc1,* known to be expressed in anti-inflammatory macrophages^42,76^ (hereafter named M2 marker). Previous studies have characterised the involvement of M1 macrophages in folliculogenesis and ovulation^34^, whereas M2 macrophages are associated with the regulation of angiogenesis^35^. We found an increase in the mRNA levels of M1 markers after 4 wk of HFD feeding but no changes in the M2 marker. Thus, dysregulation of M1 macrophages in the ovary may impair folliculogenesis and adequate oocyte development^34,77^. Additionally, our LEPT mouse model evidenced the crosstalk between leptin signalling and putative M1 macrophage regulation in the ovaries, which corroborated similar observations at 4 wk of HFD feeding, with the upregulation of M1 markers. Previous reports have established the association between leptin signalling and macrophage activation and polarisation through metabolic and immunological changes that support the desired macrophage phenotype^78^. Han et al. (2022) also showed that leptin activated the NLRP3 inflammasome and promoted M1 polarisation by the NLRP3 inflammasome^40^. Hence, our results corroborated previous observations, evidencing the importance of active leptin signalling for the regulation of marker genes for the infiltration of M1 macrophages, along with NLRP3 inflammasome activation.

Next, we analysed the transcriptional profile of GCs collected from genetically obese mice. We confirmed that despite the phenotypic similarities between the *db/db* and *ob/ob* models, profound differences characterised the transcriptional programme of GCs in homozygous mice in both strains. In *db/db* mice, the downregulated genes were linked to steroid metabolism and prostaglandin action, denoting a clear dysregulation in hormonal coordination of follicular growth. Furthermore, the upregulation of *Pdgfrb* might indicate a compensatory response associated with the disruption of follicular developmental progression. In fact, PDGFRB is the GC receptor known to modulate the signal of the oocyte secreted factor PDGF-BB, and the complex has been described as a major component controlling follicular growth^64,65^. Overall, follicular growth seemed to arrest at the antral stage, as shown by the microscopic sections of *db/db* mice. Concerning the *ob/ob* mice, genes regulating ECM and ovulation were consistently downregulated, along with genes from the *Hox* family, which were previously associated with follicular growth. Finally, a contrasting profile was observed for steroidogenesis regulation, with *db/db* mice showing a consistent downregulation of genes controlling steroidogenesis in GCs, whereas *ob/ob* mice revealed an upregulation of the same genes. This observation substantiates previous findings supporting the inhibitory role of leptin on steroidogenesis^79^ and the potential inhibition of the ovulatory process^80^, since the *db/db* mice present hyperleptinaemia, whereas *ob/ob* mice lack leptin^71^. Other studies have shown increased aromatase activity (*Cyp17a1*) in endocrine theca cells of *ob/ob* and *db/db* mice, a feature also observed in DIO models^81^. Despite presenting divergent genes, both models showed major disruptions in steroidogenesis and pathways controlling follicular growth and expansion and potentially ovulation.

Another important observation in our work was the lack of changes in the proinflammatory response in GCs from genetically obese mice, as no differences were found in either NLRP3 inflammasome gene expression or markers of macrophage infiltration. Based on previous studies undertaken in the ovary and other organs, we selected the *chemokine *(*C-X3-C motif*)* ligand 1* (*Cx3cl1*), *chemokine *(*C-X-C motif*)* ligand 1* (*Cxcl1*), *Cxcl16, Cxcl5, Il-18*, and *Il-23a* genes as M1 markers^82,83^. For M2 macrophage markers, we selected *chemokine *(*C–C motif*)* ligand 17* (*Ccl17*), *Il-4*, and *transforming growth factor, beta 1* (*Tgfb1*) ^84^, while markers of macrophage M2 infiltration comprised *Ccl25*, *colony stimulating factor 1 *(*Csf1*), *Cxcl12*, *intercellular adhesion molecule 1* (*Icam1*), *inhibin beta-B* (*Inhbb*), *macrophage migration inhibitory factor* (*Mif*), and *retinoic acid receptor responder protein 2* (*Rarres2*)^82,85–87^. Overall, none of the selected factors were differentially regulated in GCs of *db/db* and *ob/ob* mice. These observations were corroborated by the localisation of the macrophage marker CD68 in the ovarian interstice and mostly in perifollicular areas. Moreover, the expression of genes associated with leptin signalling in the GCs was unchanged in mutants (db/db and ob/ob) comparing to control mice, nor was there any difference in the expression of genes regulating NLRP3 inflammasome activation. Previous studies have reported that ovulation involves many inflammatory genes in GCs, without specific leucocyte markers^88^. Additionally, GCs can produce chemokines such as monocyte chemoattractant protein-1 (MCP-1), which has been shown to regulate follicular development and ovulation. However, high levels of MCP-1 can also be associated with inflammatory conditions such as polycystic ovary syndrome (PCOS). Furthermore, NLRP3 protein expression was shown to be upregulated in preovulatory follicles^31^. The present lack of changes in the expression of genes regulating the immune-mediated response, as well as those in leptin signalling in GCs of *ob/ob* and *db/db* mice may result in impaired late folliculogenesis and failed ovulation. These results suggest that the absence of changes in leptin signalling, alongside with NLRP3 inflammasome activation in GCs from genetically obese mice, fails to promote the macrophage phenotype switch or infiltration of macrophages and that there are other factors that can modulate their function in late folliculogenesis.

## Conclusion

In summary, our work shows the three-armed relationship among leptin signalling, NLRP3 inflammasome activation and the expression of M1 macrophages in the ovaries of obese mice. In early obesity, leptin signalling regulates NLRP3 inflammasome activation that supports M1 macrophage infiltration. Conversely, in late obesity, after the establishment of leptin resistance at 16 wk of HFD feeding, the disruption of canonical and noncanonical leptin signalling leads to failure in NLRP3 inflammasome activation and M1 macrophage regulation. Finally, the aforementioned relationship was not observed in GCs of genetically obese mice, as genes regulating leptin signalling, NLRP3 inflammasome activation or infiltration of M1 macrophages were unchanged. Nonetheless, transcriptomic analysis revealed changes in steroidogenesis, prostaglandin action and genes regulating ECM, which are known to critically regulate follicular growth. Thus, the present findings suggest a critical role of leptin signalling in the regulation of NLRP3 inflammasome activity and M1 macrophage infiltration, particularly in the theca/stromal ovarian compartment of obese mice.

### Supplementary Information

Supplementary Figure 1.

Supplementary Figure 2.

Supplementary Figure 3.

Supplementary Table 1.

Supplementary Table 2.

Supplementary Table 3.

Supplementary Table 4.

Supplementary Table 5.

Supplementary Table 6.

Supplementary Table 7.

## Data Availability

Datasets we submitted to Gene Expression Omnibus with the number GSE236196.
