# Supplementary figures and images for: Leptin signalling regulates transcriptional differences in granulosa cells from genetically obese mice but not the activation of NLRP3 inflammasome
Source: Sci Rep. 2024 Apr 5;14:8070. doi: 10.1038/s41598-024-58181-w (PMC10997671; doi:10.1038/s41598-024-58181-w)

A

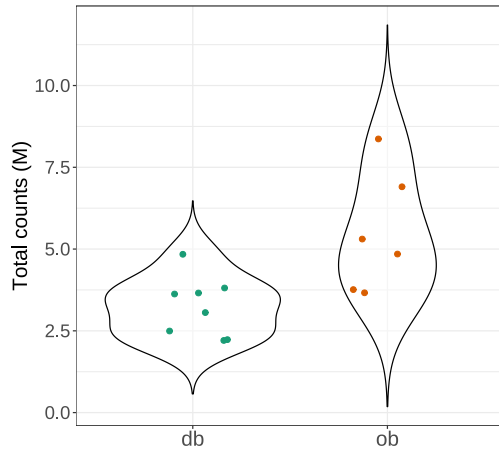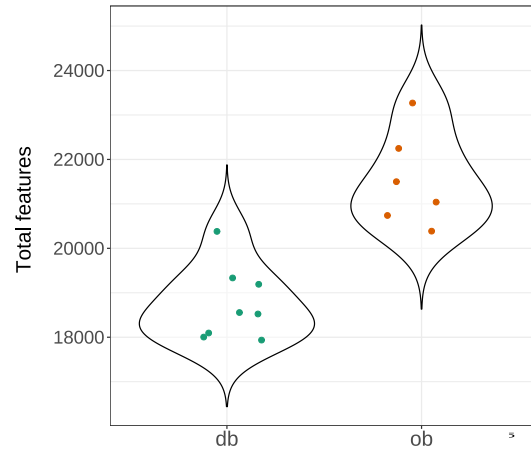

B

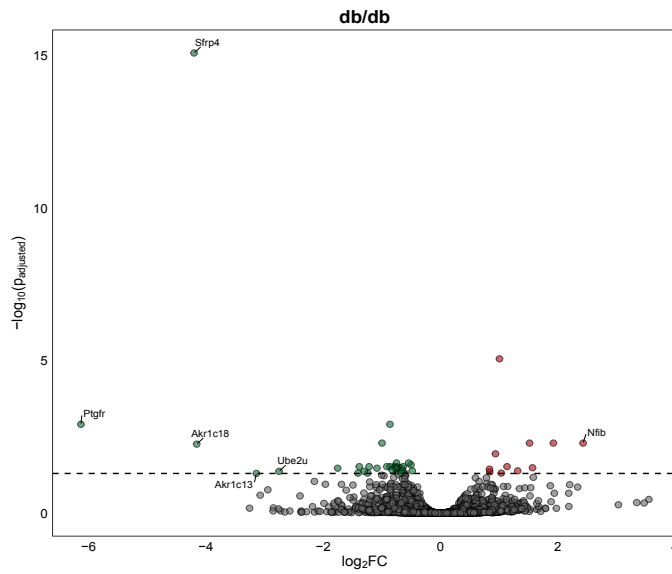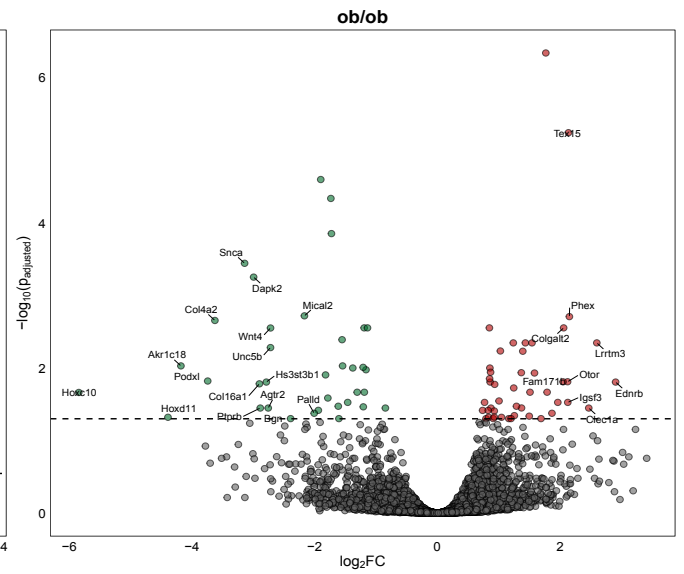

C

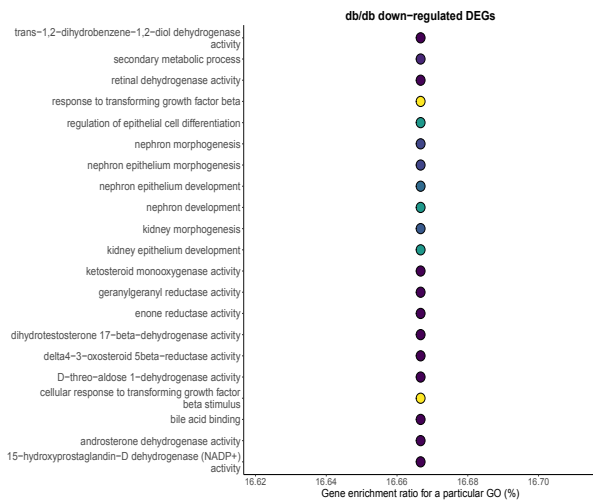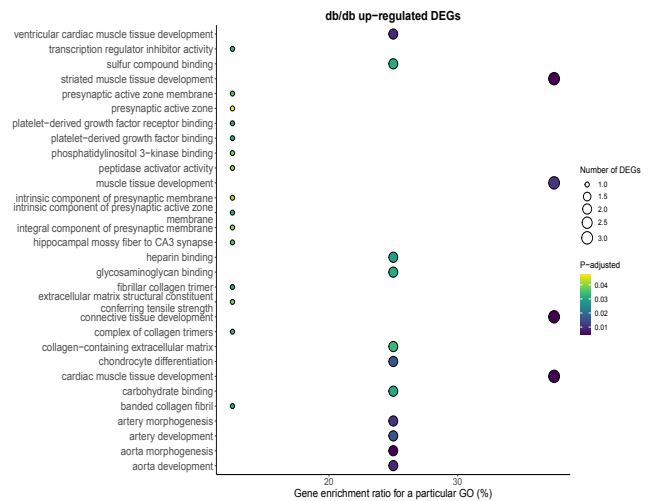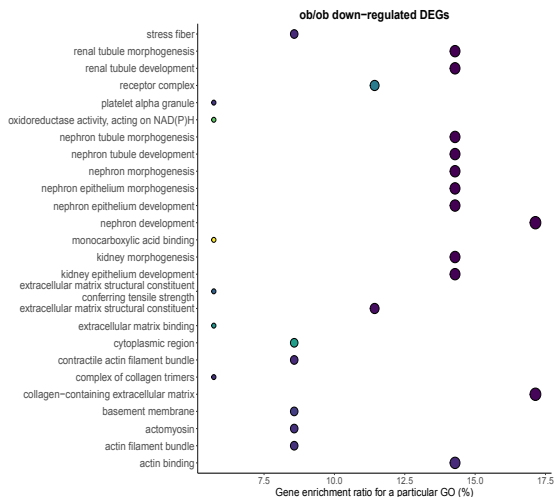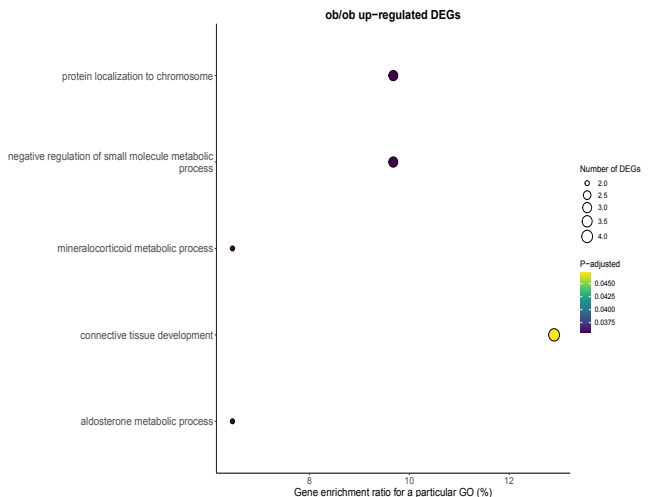

Supplement: Supplementary file 1 — Supplementary Figure 1. [file 41598_2024_58181_MOESM1_ESM.pdf]

## A NLRP3 for db/db

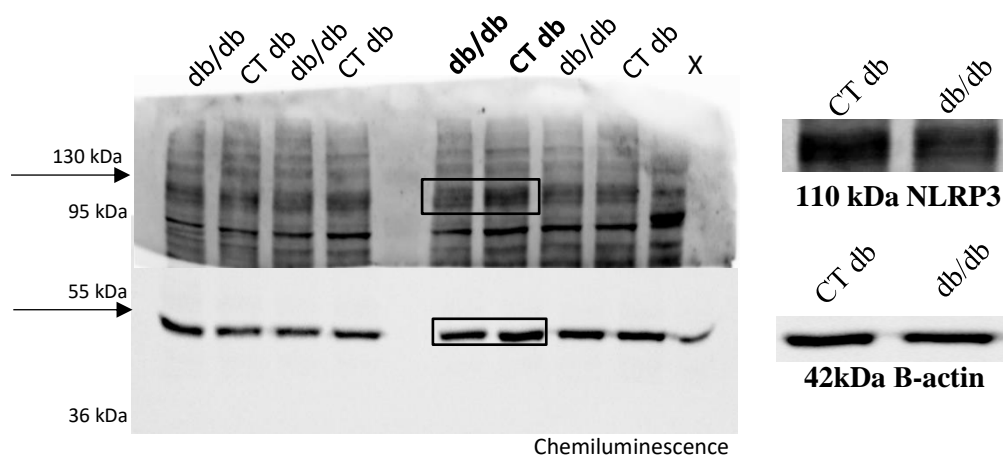

## B CASP1 for db/db

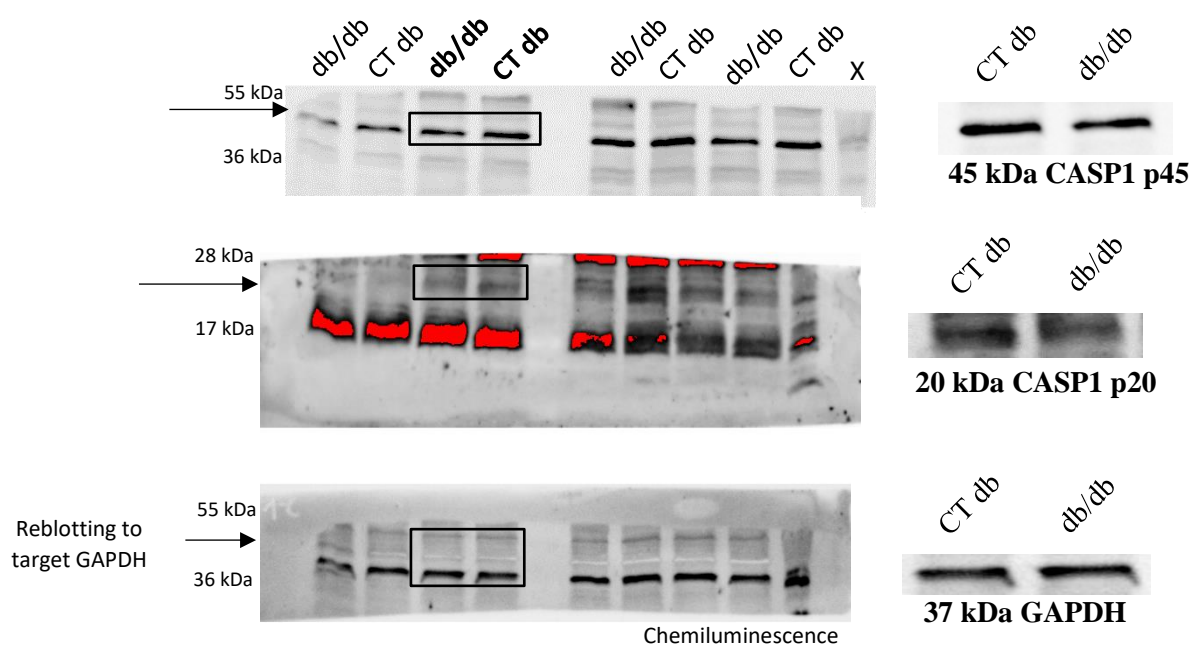

## C IL-18 for db/db

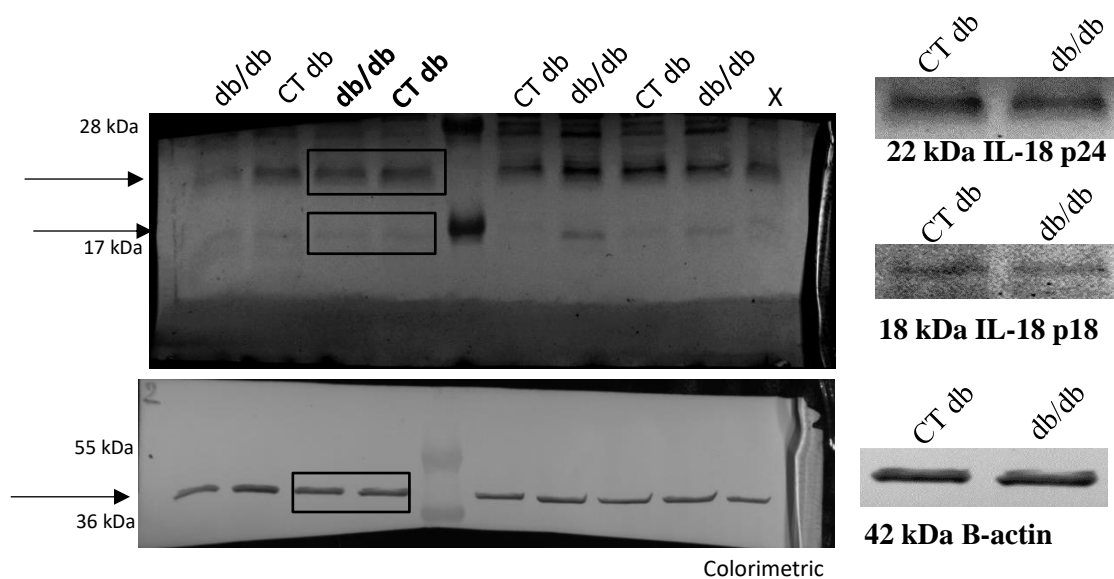

Supplement: Supplementary file 2 — Supplementary Figure 2. [file 41598_2024_58181_MOESM2_ESM.pdf]

## A NLRP3 for DIO

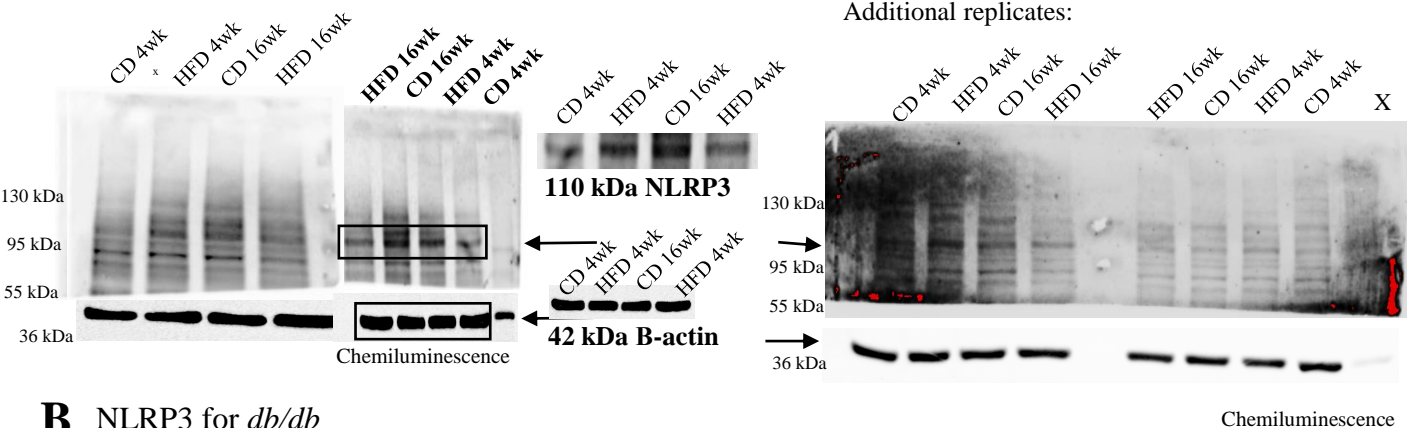

## B NLRP3 for *db/db*

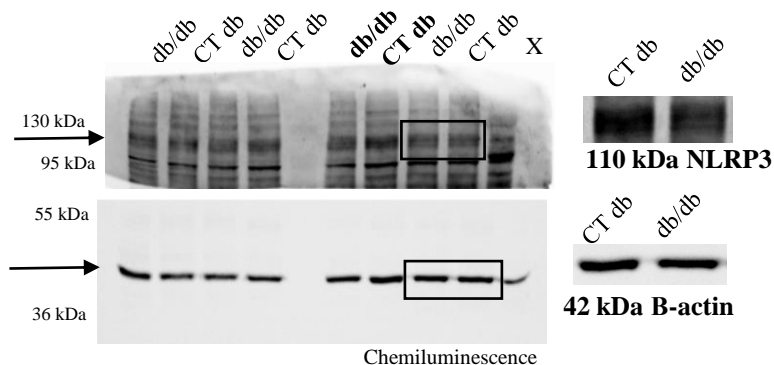

## C NLRP3 for *ob/ob*

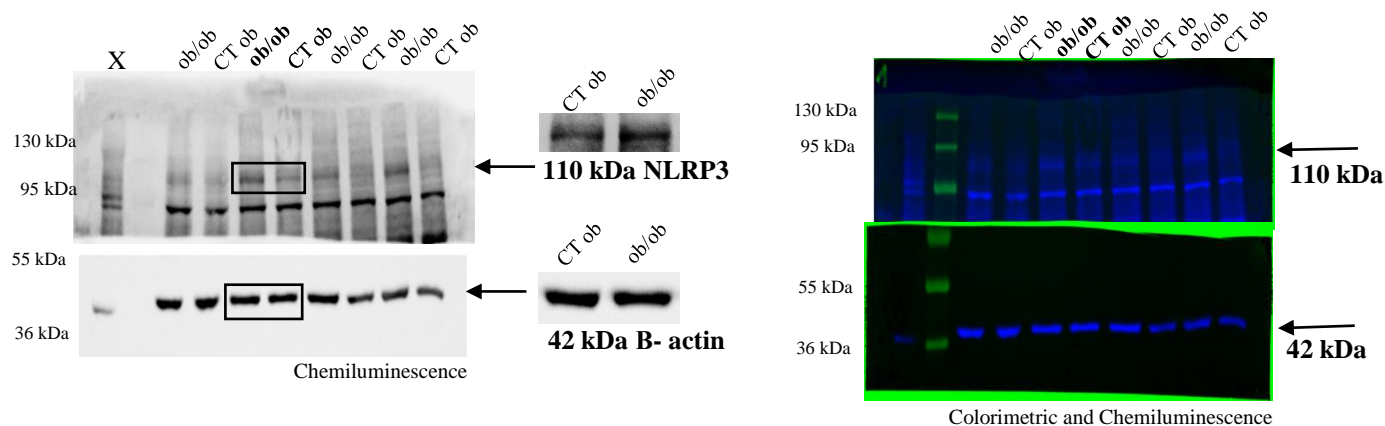

## D NLRP3 for LEPT

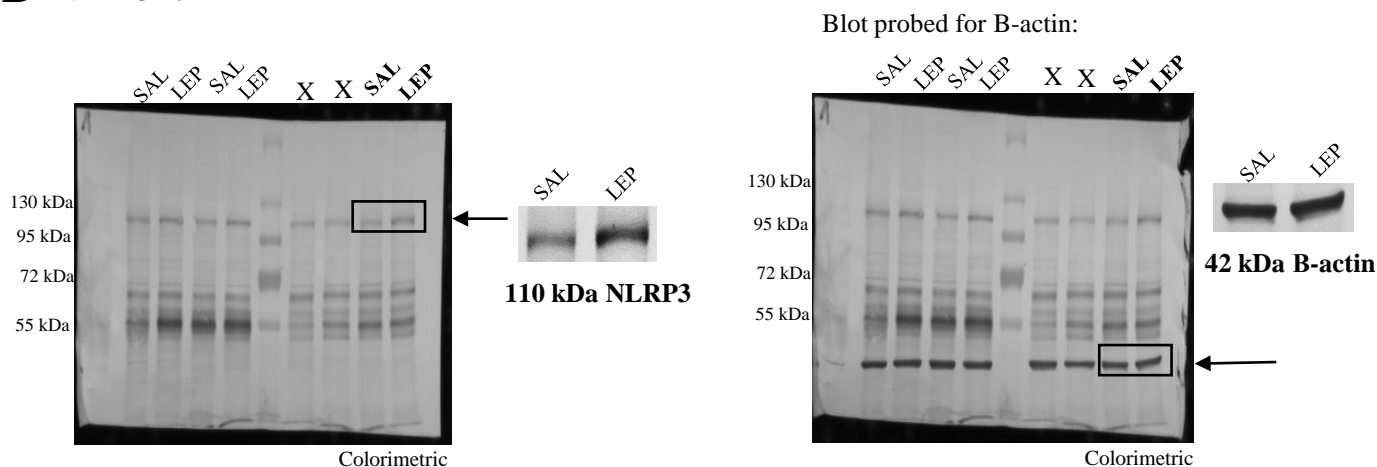

Supplement: Supplementary file 3 — Supplementary Figure 3. [file 41598_2024_58181_MOESM3_ESM.pdf]
